# Depressed mood in pregnancy: Prevalence and correlates in two Cape Town peri-urban settlements

**DOI:** 10.1186/1742-4755-8-9

**Published:** 2011-05-02

**Authors:** Mary Hartley, Mark Tomlinson, Erin Greco, W Scott Comulada, Jacqueline Stewart, Ingrid le Roux, Nokwanele Mbewu, Mary Jane Rotheram-Borus

**Affiliations:** 1 Department of Psychology, Stellenbosch University, Private Bag X1, Matieland, 7602, South Africa; 2 Semel Institute for Neuroscience and Human Behavior, Center for Community Health, University of California, Los Angeles, USA; 3 Philani Child Health and Nutrition Project, Khayelitsha, PO Box 40188, Elonwabeni, Cape Town, 7791, South Africa; 4 Woodrow Wilson School of Public & International Affairs, Princeton University, Princeton, NJ 08544, USA

## Abstract

**Background:**

The disability associated with depression and its impact on maternal and child health has important implications for public health policy. While the prevalence of postnatal depression is high, there are no prevalence data on antenatal depression in South Africa. The purpose of this study was to determine the prevalence and correlates of depressed mood in pregnancy in Cape Town peri-urban settlements.

**Methods:**

This study reports on baseline data collected from the Philani Mentor Mothers Project (PMMP), a community-based, cluster-randomized controlled trial on the outskirts of Cape Town, South Africa. The PMMP aims to evaluate the effectiveness of a home-based intervention for preventing and managing illnesses related to HIV, TB, alcohol use and malnutrition in pregnant mothers and their infants. Participants were 1062 pregnant women from Khayelitsha and Mfuleni, Cape Town. Measures included the Edinburgh Postnatal Depression Scale (EPDS), the Derived AUDIT-C, indices for social support with regards to partner and parents, and questions concerning socio-demographics, intimate partner violence, and the current pregnancy. Data were analysed using bivariate analyses followed by logistic regression.

**Results:**

Depressed mood in pregnancy was reported by 39% of mothers. The strongest predictors of depressed mood were lack of partner support, intimate partner violence, having a household income below R2000 per month, and younger age.

**Conclusions:**

The high prevalence of depressed mood in pregnancy necessitates early screening and intervention in primary health care and antenatal settings for depression. The effectiveness and scalability of community-based interventions for maternal depression must be developed for pregnant women in peri-urban settlements.

**Trial registration:**

ClinicalTrials.gov: NCT00972699.

## Background

Depression is a leading cause of disability worldwide [1]. Despite its high prevalence and known correlation with poverty [2], data for low and middle income (LAMI) countries is limited. Mental health is neglected in the national policies of many LAMI countries [3], and is of critical public health significance because of its intergenerational impact on infants and children as a result of its impact on disease burden and child health. Regardless of income, postnatal depression negatively affects child development and the mother-infant relationship [4,5]. In LAMI countries, it is also associated with poor child growth [6,7]; poor mental development [7]; and higher risk for infant diarrhoea [6]; Postnatal depression is associated with maternal disability, which affects the care giving capacity of mothers for their infants [8]. In LAMI countries, where circumstances such as overcrowding, food insecurity and poor sanitation are commonplace, this sub-optimal care from the mother has detrimental effects for the health of her child [3].

Though less well documented than postpartum depression, depression in pregnancy is also associated with adverse child outcomes. Depression places women at greater risk for inadequate prenatal care, alcohol use and poorer weight gain in pregnancy: each of these factors affects the unborn infant [9]. Depression in pregnancy is associated with spontaneous pre-term births [10]; slower foetal growth [11]; with depressed infant behaviour in general [12]; and with increased incidence of depression in infants when they are adolescents [13]. Other studies have not found an association between depression during pregnancy and adverse obstetric outcomes [14,15], while a recent review has shown that women with depression during pregnancy are at increased risk for pre-term birth and low birth weight [16]. Depression is also a strong predictor of postnatal depression, with women who are depressed in pregnancy having a heightened risk of developing depression during the postpartum period [9,17-19]. Research from Ethiopia has found Common Mental Disorders (CMD) in pregnancy, which are characterised by depressive, anxious, panic and somatic symptoms, to be associated with prolonged labour (of more than 24 hours), delayed initiation of breastfeeding, and more diarrhoeal episodes [20].

Although the prevalence rates for postpartum depression in South Africa are high (34.7%) [5], there are no prevalence data on antenatal depression. The detection of antenatal depression is important in that it is a predictor of postnatal depression [21] and it has been shown that it can be treated and done so in a cost effective manner [22]. The present study aimed to determine the prevalence of depressed mood in pregnancy in Cape Town peri-urban settlements, and to identify risk factors associated with it in this population.

## Methods

### Sample

This study reports on baseline data collected from the Philani Mentor Mothers Project (PMMP), a community-based, cluster-randomized controlled trial currently underway in Khayelitsha and Mfuleni in Cape Town, South Africa. All pregnant women in 24 neighbourhoods were approached to participate in a longitudinal study of family health. If nobody was found at home during an initial recruitment visit, recruiters would continue to visit the household until somebody was present to ensure that no pregnant women were missed. The present study used baseline data from the first 1062 participants. The sample was generated by neighbourhood recruiters who went door to door in each study neighbourhood, introducing the study to all households, and asking about any pregnancies. When a pregnant woman over the age of 18 years was found, she was invited to participate in the study.

### Instruments

Depressed mood was determined with a commonly used screening instrument, the Edinburgh Postnatal Depression Scale (EPDS) [23]. The EPDS has been validated for use in pregnancy in both high income [24], and LAMI countries [25,26]. The scale consists of 10 items pertaining to the common mood characteristics of depression experienced in the past week. It takes approximately five minutes to administer, and each item is scored on a continuum of 0-3, allowing a total score between 0 and 30, where a higher score indicates greater distress. Research has supported the construct validity of an interviewer-administered isiXhosa version of the EPDS for use in South Africa [27]. The EPDS has also demonstrated satisfactory internal reliability - a cronbach alpha coefficient of 0.89 [27]. In South Africa, there have been two validation studies of the EPDS in community samples. The first found an optimal threshold of 11/12, or 12 and above, for women in the postnatal period [28]. The second (manuscript in preparation) found that that a threshold of 13/14, or 14 and above, was optimal for classifying 'probable' cases of depression [26]. The present study uses this threshold as a basis for interpretation.

Socio-demographic variables collected included maternal age, household income, parity, education, marital status, relationship violence experienced in the previous year (including any kind of violence - pushing/shoving, being slapped/punched, having a weapon used against one), if the baby was planned, financial support from the baby's father, smoking during pregnancy, and perceived social support (with regards to the woman's partner, mother and father). Social support questions were derived from methods used by Cooper et al. (1999) [5].

Alcohol use was assessed using the Derived Alcohol Use Disorder Identification Test from the National Epidemiologic Survey on Alcohol and Related Conditions (Derived AUDIT-C) [29]. The Derived AUDIT-C is a three-item questionnaire based upon the original 10-item AUDIT [30], which has been used extensively to assess alcohol use in both men and women in the Cape Town region of South Africa [31]. The Derived AUDIT-C is highly correlated with the original AUDIT [29] but includes modifications to the first three questions and is based solely on items reflecting alcohol consumption. The tool was developed to meet the challenge of brevity and ease of administration in busy clinics. The three questions on the screen include: (1) days of any alcohol use; (2) usual number of drinks per day; and (3) binge episodes of five or more drinks in a single day. For this study, question 3 was modified to define a binge episode as heavy episodic drinking of four or more drinks in a single day. Acknowledgment of any alcohol use post conception classified the woman as drinking during pregnancy.

### Procedure

All pregnant women over the age of 18 were collected from their homes and driven to the research centre located in Khayelitsha, Cape Town. Following informed consent, participants were interviewed using a structured questionnaire which was pre-programmed into a mobile phone. Data collectors, who were women fluent in both isiXhosa and English, read the questions from the mobile phone and participants' responses were then entered into the phones. The use of mobile technology in data collection allows for simple logic and range validation to be performed as questions are asked, which contributes to improved data quality. Confidentiality is also maximised by the mobile technology as the data is encrypted, and uploaded to a central database which is protected by firewalls as soon as network reception is identified. As the data is uploaded, it is automatically deleted from the phone. After each interview, participants were given a food voucher to the value of R80 as a participation incentive, and then driven home. Interviews lasted an average of one hour. The protocol for this study was approved by the Health Research Ethics Committee of Stellenbosch University (N08-08-218), and the Institutional Review Board at the University of California at Los Angeles (G07-02-033).

### Statistical analysis

Data analysis was conducted using SAS software version 9.2 (SAS Institute Inc., Cary, NC, USA). Descriptive data on the total sample were first examined. Pregnant women were then classified as having depressed mood or not, based on a score greater than or equal to 14 on the EPDS. Bivariate comparisons of groups were performed using Chi-square analysis for categorical variables, and Wilcoxon-Mann-Whitney tests for continuous variables because these were not normally distributed. Logistic regression was then performed including the variables that had a significant (p < 0.05) bivariate relationship with EPDS as well as demographic variables (age, education, formal housing, services, and weeks pregnant). Regression diagnostics for outliers, error residuals and multicolinearity were assessed.

## Results

### Socio-demographic characteristics

Of 1069 women invited to participate in the study, seven refused, resulting in a total sample of 1062 participants. The mean age of the sample was 26 years (SD = 5.5). Most participants were in either their second (46%) or third (48%) trimester of pregnancy. Thirty-eight percent of the sample were primiparous, and 42% percent of women were married or cohabiting with a partner. Twenty-six percent had completed secondary schooling, and 8% had completed no formal education beyond primary school. The socio-economic circumstances of women in the sample were poor. More than half of the sample (54.4%) reported a household income of below R2000 per month, and 80.7% of the participants were unemployed. More than two thirds (69.1%) of women lived in informal housing (made without foundation from corrugated iron, wood, plastic and other waste materials), and 45.9% of women had access to either none or only one of the following services: water on the premises, a flush toilet on the premises, and electricity on the premises. Pregnancies were unplanned in 73.3% of the sample.

### Prevalence of depressed mood

Depressed mood in pregnancy was reported by 39% of mothers. The EPDS demonstrated good internal reliability, with a cronbach's alpha of 0.87.

### Correlates of depressed mood

Bivariate comparisons are presented in Table 1 and 2. Factors which were significantly associated with depressed mood at a p < 0.05 level included being single as opposed to being married or cohabiting with a partner, being unemployed, having a household income below R2000 per month, having less education, smoking, alcohol use, experiencing intimate partner violence in the previous year, receiving poorer social support from one's partner, mother and father, and receiving no financial support from the baby's father.

**Table 1:** Bivariate comparisons for dichotomous variables (N = 1062)

	EPDS 0-13		EPDS 14-30				
	N = 652 (61%)		N = 410 (39%)			95% CI	
	n	%	n	%	OR	Lower	Upper
Marital Status							
Single	255	39.1%	196	47.8%	1.43	1.11	1.83**
Married/cohabitating	397	60.9%	214	52.2%			
							
Housing type							
Informal	442	67.8%	292	71.2%	1.18	0.90	1.54
Formal	210	32.2%	118	28.8%			
							
Employment							
Unemployed	511	78.4%	346	84.4%	1.49	1.08	2.07*
Employed	141	21.6%	64	15.6%			
							
Household Income							
R0-R2000	312	48.8%	247	63.7%	1.84	1.42	2.39**
R2001+	328	51.3%	141	36.3%			
							
Financial support baby father							
No	84	12.9%	99	24.3%	2.16	1.57	2.99**
Yes	567	87.1%	309	75.7%			
							
Smoking							
No	633	97.1%	388	94.6%			
Yes	19	2.9%	22	5.4%	1.89	1.01	3.54*
							
Alcohol use							
No	495	75.9%	272	66.3%			
Yes	157	24.1%	138	33.7%	1.60	1.22	2.10**
							
Baby planned							
No	464	71.3%	313	76.5%	1.31	0.99	1.75
Yes	187	28.7%	96	23.5%			
							
Partner violence							
No	435	66.7%	221	53.9%			
Yes	217	33.3%	189	46.1%	1.71	1.33	2.21**
							

*p < 0.05, **p < 0.01

**Table 2:** Bivariate comparisons for continuous variables (N = 1062)

	EPDS 0-13		EPDS 14-30		
	N = 652 (61%)		N = 410 (39%)		Wilcoxan
	Mean	SD	Mean	SD	Z approx
Age	26.6	5.6	26	5.3	-1.48
Education	10.5	1.8	10.1	1.9	-3.51**
Services	2.0	1.1	1.9	1.2	-1.06
Partner Support	4.1	1.7	3.3	1.9	-6.44**
Mother Support	3.9	2.2	3.4	2.3	-3.40**
Father Support	1.6	1.9	1.3	1.8	-2.75**
Previous Children	0.99	1.0	0.99	1.1	-0.20
Weeks pregnant	25.8	8.0	26.0	8.4	0.78

*p < 0.05, **p < 0.01

Results from the logistic regression are presented in Table 3. Higher odds of depressed mood were associated with less partner support (OR = 0.88, 95% CI = 0.8-0.97), relationship violence in the previous year (OR = 1.49, 95% CI = 1.13-1.96), having a household income of below R2000 per month (OR = 1.52, 95% CI = 1.15-2.01), and younger age (OR = 0.97, 95% CI = 0.95-1.0). No other variables remained significant in the multivariate model.

**Table 3:** Logistic regression analysis: Predictors of depressed mood (N = 1062)

Predictor	Odds Ratio	95% Confidence Interval	
Age	0.97	0.95	1.00*
Education	0.93	0.87	1.01
Services	0.98	0.84	1.14
Partner support	0.88	0.80	0.97**
Mother support	0.95	0.89	1.01
Father support	0.95	0.88	1.02
Weeks pregnant	1.01	0.99	1.02
Single vs. married/living together	0.93	0.68	1.28
Informal housing vs. formal	1.04	0.72	1.49
Unemployed vs. employed	1.09	0.76	1.56
HH income < R2000 p/m	1.52	1.15	2.01**
No income baby father	1.20	0.78	1.83
Tobacco use	1.33	0.67	2.65
Alcohol use	1.22	0.89	1.66
Relationship violence	1.49	1.13	1.96**

*p < 0.05, **p < 0.01

## Discussion and conclusions

Results endorse findings from other LAMI countries that the prevalence of depressed mood is higher in economically deprived populations than in rich contexts. Depressed mood was present for 39% of women in the present study, compared with 7.4% to 12.8% found in high income countries, depending on the trimester of pregnancy [32]. It is also higher than the prevalence found in several other LAMI countries such as Nigeria, Pakistan and Brazil, where prevalence rates are 10.8% [33], 25% [34] and 20% [35] respectively. The prevalence for postnatal depression in Cape Town peri-urban settlements is 34.7% [5], suggesting that the prevalence of distress throughout the time surrounding childbirth is high.

The strongest predictors of depressed mood in pregnancy were lack of emotional support from women's partners, relationship violence, a household income below R2000 per month, and young age. The association between poor partner support and maternal depression is consistent with research from many countries both pre and postnatally [36-39]. Similarly, research from several LAMI countries finds violence to be associated with depressed mood both in pregnancy [35,40], and in the postnatal period [8]. In South Africa, this association is concerning because domestic violence against women is highly prevalent [41], and especially so in populations where poverty is endemic [42].

The association between household income and depressed mood is evidence of a relationship between economic deprivation and depression. Although no longer significant after controlling for other variables, being unemployed, being poorly educated and receiving no financial support from the baby's father were also associated with depressed mood in the bivariate analysis. Housing type as formal or informal and household services, however, were not. It might be that because economic disadvantage was endemic to the entire population, that we were not fully able to examine the role of these variables.

Having an unplanned pregnancy was not associated with depressed mood in pregnancy, although it has been found to be associated with postnatal depression in South African peri-urban settlements [37]. Smoking in pregnancy and alcohol use were not associated with depressed mood in the multivariate model, although both reached significance in the bivariate analysis. This is consistent with research from several countries, where an association between depression and substance abuse is well documented [32,40]. In addition to Fetal Alcohol Syndrome, co-morbid alcohol use and mental disorders have been shown to have other negative consequences for infant health, with women diagnosed with co-morbid substance use disorders and psychiatric disorders being more likely to deliver low birth weight and preterm infants than those with either of these conditions alone [43]. Furthermore, women with higher levels of depression often continue to use alcohol despite knowing they are pregnant and clinician advice against such use [44], which has critical implications for infant health in South Africa where we have the highest rate of Fetal Alcohol Syndrome in the world [45,46]. Smoking in pregnancy also places unborn infants at greater risk for late foetal and neonatal mortality, and low birth weight [47]. The crude association between substance use and depressed mood supports the argument that effective treatment of co-occurring conditions should involve the integration of mental health and substance abuse treatment services in a cohesive and unitary system of care [48].

To the best of our knowledge, this is the first study in South Africa to examine the prevalence and correlates of depressed mood in pregnancy. However, several important limitations should be noted. This study lacked clinical validation of the EPDS, and is therefore subject to error that arises from false positives and negatives inherent when using screening tools. The cross sectional design of this study does not allow us to ascertain causality, and longitudinal prospective research is needed in South Africa to fully understand the nature of social factors in antenatal depression, and the impact of antenatal depression on maternal and child health. Future research might examine threatening life events and extreme societal stressors, which were not investigated in the current study, but have been found to influence maternal depression [36,49]. Finally, research examining the relationship between antenatal depression and child health in South Africa is needed.

The WHO has advised that health policy integrate mental health care into primary health care settings [50]. Although further research is needed to establish the scalability and effectiveness of interventions for depression in community contexts, this study provides an important step in documenting the need for antenatal screening for depression. Pregnancy is a time in many women's lives when they are most likely to access the health system by way of antenatal care, and is therefore a plausible time to implement screening and intervention. Given the high prevalence of antenatal distress, early intervention may have important child health implications. Antenatal depression heightens the risk of postpartum depression, and both antenatal and postnatal depression impact on child outcomes. While maternal mental health is currently a low priority in the health care practises of most LAMI countries, the findings of this paper highlight the importance of addressing mental health in antenatal care.

## Competing interests

The authors declare that they have no competing interests.

## Authors' contributions

MH drafted the first version of the paper, conducted statistical analysis and supervised data collection. MT designed the study and has taken a major role in writing the submitted paper. EG did the statistical analysis of the data and played an important role in writing the manuscript. WSC contributed to design of the study, to drafting and critically revising the manuscript and conducted statistical analysis. JS and NM contributed to the acquisition of data, and to drafting and critically revising the manuscript. MRB designed the study, acquired funding for the study and has taken a major role in writing the submitted paper. All authors reviewed and approved the final version of the manuscript.
